# Quantitative trait locus analysis of parasitoid counteradaptation to symbiont-conferred resistance

**DOI:** 10.1038/s41437-021-00444-7

**Published:** 2021-05-19

**Authors:** Gabriel F. Ulrich, Niklaus Zemp, Christoph Vorburger, Hélène Boulain

**Affiliations:** 1 EAWAG, Swiss Federal Institute of Aquatic Science and Technology, Überlandstrasse 133, 8600 Dübendorf, Switzerland; 2 Institute of Integrative Biology, ETH Zürich, Universitätsstrasse 16, 8092 Zürich, Switzerland; 3 Genetic Diversity Centre, Department of Environmental Systems Sciences, ETH Zürich, 8092 Zürich, Switzerland; 4 Present Address: Department of Ecology and Evolution, University of Lausanne, 1015 Lausanne, Switzerland

**Keywords:** Experimental evolution, Evolutionary genetics, Evolutionary ecology, Genetic linkage study

## Abstract

Insect hosts and parasitoids are engaged in an intense struggle of antagonistic coevolution. Infection with heritable bacterial endosymbionts can substantially increase the resistance of aphids to parasitoid wasps, which exerts selection on parasitoids to overcome this symbiont-conferred protection (counteradaptation). Experimental evolution in the laboratory has produced counteradapted populations of the parasitoid wasp *Lysiphlebus fabarum*. These populations can parasitize black bean aphids (*Aphis fabae*) protected by the bacterial endosymbiont *Hamiltonella defensa*, which confers high resistance against *L. fabarum*. We used two experimentally evolved parasitoid populations to study the genetic architecture of the counteradaptation to symbiont-conferred resistance by QTL analysis. With simple crossing experiments, we showed that the counteradaptation is a recessive trait depending on the maternal genotype. Based on these results, we designed a customized crossing scheme to genotype a mapping population phenotyped for the ability to parasitize *Hamiltonella*-protected aphids. Using 1835 SNP markers obtained by ddRAD sequencing, we constructed a high-density linkage map consisting of six linkage groups (LGs) with an overall length of 828.3 cM and an average marker spacing of 0.45 cM. We identified a single QTL associated with the counteradaptation to *Hamiltonella* in *L. fabarum* on linkage group 2. Out of 120 genes located in this QTL, several genes encoding putative venoms may represent candidates for counteradaptation, as parasitoid wasps inject venoms into their hosts during oviposition.

## Introduction

Interactions between organisms are important drivers of evolution. Among them, host–parasite interactions represent particularly intimate relationships between species (Windsor 1998). In this context, reciprocal selection can lead to antagonistic coevolution through dynamic adaptation and counteradaptation, often compared to an arms race between species (Van Valen 1977; Woolhouse et al. 2002). Classical models of host–parasite coevolution treat resistance and infectivity as traits encoded by the genome of the host and parasite, respectively (Anderson and May 1982; Frank 1997; Sasaki 2000). While this is often the case (e.g., Flor 1956; Dubuffet et al. 2007; Bento et al. 2017), there are also numerous examples where these traits are influenced or even determined by symbionts associated with hosts or parasites. For example, the human gut microbiome is an important determinant of susceptibility to pathogens (Bäumler and Sperandio 2016), microbial endosymbionts can increase insect resistance to various types of parasites (Oliver et al. 2014), and even macrobial symbionts like tending ants can reduce parasitism of honeydew-producing insects (Itioka and Inoue 1996). Similarly, parasites may be aided by symbionts for successful infection, as in the case of insect-parasitic nematodes and their symbiotic *Xenorhabdus* and *Photorhabdus* bacteria (Goodrich‐Blair and Clarke 2007), or in parasitic wasps aided by viral symbionts (Coffman and Burke 2020).

In the case of maternally transmitted endosymbionts protecting hosts against parasitism (Haine 2008), the symbiont’s contribution to host resistance is heritable and responsive to selection by parasites (Jaenike 2012). As a result, the reciprocal adaptation in host–parasite coevolution becomes mediated by symbionts (reviewed in Vorburger and Perlman 2018). For these adaptive changes to happen, genetic variation is required in both parasite and host populations. The advent of next-generation sequencing combined with laboratory-based experiments manipulating host–parasite systems has allowed to investigate this genetic diversity and address the genomic basis underpinning the mechanisms of coevolution (Schlötterer et al. 2015).

Parasitoids form a peculiar group within parasites as they inevitably kill their hosts upon completion of their life cycle (Godfray 1994). Parasitoids thus tend to have more severe impacts on host fitness than parasites, imposing strong selection on the host to resist. The large majority of parasitoid insects belongs to the order Hymenoptera, which comprises at least several hundred thousand species of parasitoid, accounting for >75% of the order’s species richness (Heraty 2009; Forbes et al. 2018). While idiobiont parasitoids kill or paralyze the host during oviposition to arrest host development, koinobiont parasitoids allow their host to continue development during parasitism (Quicke 2015). Hence, koinobiont endoparasitoids are exposed to and must bypass the host’s immune system while keeping it functional to prevent the host from succumbing to opportunistic infections. During koinobiont evolution, various strategies have therefore been selected to finely manipulate the host’s immune defenses (Pennacchio and Strand 2006; Burke and Strand 2014).

Aphids and their associated endoparasitoid wasps provide a unique system to study host–parasite coevolution as, in many cases, the outcome of their interaction is also influenced by facultative bacterial endosymbionts of aphids (Oliver et al. 2003). The vertically transmitted *Hamiltonella defensa* (Enterobacteriaceae) is among the most widespread of these symbionts (Oliver et al. 2003; Moran et al. 2005)—being found in 41% out of 131 examined aphid species (Henry et al. 2015). It confers significant protection against parasitoid wasps in multiple aphid species (reviewed in Oliver et al. 2014; Vorburger 2014). The protection is associated with the presence of the APSE bacteriophage in *H. defensa*’s genome (Oliver et al. 2009). Indeed, APSE carry strain-specific “cassettes” encoding toxins likely interfering with parasitoid development (Oliver et al. 2009). However, resistance against parasitoids is not only attributable to the symbiont–bacteriophage association aphids carry, as different symbiont-free aphid genotypes also differ in their resistance (Sandrock et al. 2010; Martinez et al. 2014). The variation in aphid susceptibility to parasitism, along with *H. defensa* strain-specific strength of protection, may explain natural variation observed in aphid–wasp interactions (Oliver et al. 2005; McLean and Godfray 2015; Oliver and Higashi 2019). Moreover, there is also ample genetic variation in wasp infectivity (Schmid et al. 2012; Vorburger and Rouchet 2016).

This natural variation is particularly well described in the interaction between the black bean aphid *Aphis fabae* (Aphididae) and the koinobiont parasitoid wasp *Lysiphlebus fabarum* (Braconidae), for which significant genotype-by-genotype interactions occur between wasp genotypes and *H. defensa* strains protecting their aphid hosts (Schmid et al. 2012; Cayetano and Vorburger 2013). A first experimental evolution study with wild-collected *L. fabarum* populations demonstrated rapid and strain-specific adaptation of parasitoids to *Hamiltonella*-protected aphids, likely due to standing genetic variation in natural populations (Rouchet and Vorburger 2014). A follow-up study also employing experimental evolution confirmed these results and compared patterns of gene expression between *Hamiltonella*-adapted parasitoids and controls using transcriptomics (Dennis et al. 2017). Starting from a genetically diverse initial wasp population, replicated subpopulations were evolved on a single *A. fabae* genotype harboring either the *H. defensa* strain H76, the *H. defensa* strain H402, or no defensive symbiont (control). While symbiont-conferred resistance was high at the beginning, wasps facing *Hamiltonella-*protected aphids exhibited significant counteradaptation (i.e., evolved ability to parasitize protected hosts) already after ten generations, while control wasps remained poorly able to parasitize aphids harboring *H. defensa*. Putative venom genes and virus-associated genes were overrepresented among the differentially expressed genes between *Hamiltonella*-adapted and control populations, suggesting a role of these genes in the counteradaptation (Dennis et al. 2017). Despite these findings, the genetic architecture and the molecular mechanisms underlying the counteradaptation remain unclear. Moreover, the contributions of larval and maternal genotypes to the counteradapted phenotype are yet to be determined.

Both maternal and larval traits can influence parasitism success in parasitoid wasps (Burke and Strand 2014). Maternal behavior may play a role in parasitoid counteradaptation, since preference for younger hosts (Schmid et al. 2012) and self-superparasitism, that is depositing more than one egg per host (Oliver et al. 2012), were both shown to affect reproductive success of wasps on protected hosts. However, there was no evidence that these traits evolved in the study by Dennis et al. (2017). Other maternal factors like venoms, polydnaviruses, and virus-like particles, which many parasitoid wasp species inject into the host alongside their eggs to circumvent host defenses, may also be involved in counteradaptation (Moreau and Asgari 2015; Dennis et al. 2017; Drezen et al. 2017). Larval traits, such as toxins or teratocyte excretion, may as well contribute to the counteradapted phenotype (Burke and Strand 2014).

Taking advantage of the recently published genome of *L. fabarum* (Dennis et al. 2020) and the continued availability of the experimentally evolved populations created by Dennis et al. (2017), we conducted a quantitative trait locus (QTL) study to investigate the genomic architecture of the counteradaptation to *H. defensa* in *L. fabarum* wasps. We first characterized the general nature of the counteradaptation trait (maternal vs larval determination, dominant vs recessive inheritance) with crossing experiments followed by parasitism bioassays. Based on these results, we designed a crossing scheme for high-density linkage map construction and QTL mapping. We used ddRAD sequencing for genotyping and measured parasitism success on *Hamiltonella*-protected hosts (offspring counts) as our phenotypic measure of parasitoid counteradaptation.

## Materials and methods

### Host and parasitoid lines

Black bean aphids (*A. fabae*) were reared on their host plant *Vicia faba* (Fabaceae) in a climate chamber at 22 °C with a 16-h photoperiod to ensure clonal reproduction. Two sublines of *A. fabae* clone A06-407 were used: the original A06-407 clone, which was free of any known defensive endosymbionts, and the modified A06-407 clone harboring the *H. defensa* strain H76 (Vorburger et al. 2009; Dennis et al. 2017). The original (*H. defensa* negative) and the modified (*H. defensa* positive, harboring the *H. defensa* strain H76) aphid lines are in the following called H− and H+, respectively.

We used two experimentally evolved populations of the parasitoid wasp *L. fabarum*. One was adapted to the presence of *Hamiltonella* in host aphids, the other was not. Wasp populations were established by Dennis et al. (2017) from a mixture of nine collections of sexually reproducing, haplo-diploid *L. fabarum* from six locations across Switzerland. Experimental evolution was conducted by rearing wasps exclusively on H− or H+ aphids, leading to counteradaptation in the H+ treatment; wasps reared on H+ aphids evolved an improved ability to parasitize H+ aphids compared to wasps reared on H− aphids (see Dennis et al. 2017 for more details). After maintaining treatments for 24 generations in 4 replicate populations each, replicates were combined and treatments were continued unreplicated at a population size of 200 individuals (see Rossbacher and Vorburger 2020 for details). Until the onset of the experiments presented here, parasitoid populations had been reared for approximately 140 generations on either H− or H+ aphids (since September 2013). At this point, the population reared on H+ aphids was able to parasitize H+ aphids nearly as well as H− aphids, whereas the population reared on H− aphids was only able to parasitize H− aphids but not H+ aphids. In the following, we refer to the wasp population adapted to H+ aphids as R ( = Resistant to *Hamiltonella*) and to the population adapted to H− aphids as S ( = Susceptible to *Hamiltonella*).

### Experiment 1: characterization of general inheritance patterns

To determine whether the evolved ability to parasitize H+ aphids is mainly determined by the larval or the maternal genotype, and whether it shows a dominant or recessive inheritance pattern, crossing experiments were combined with no-choice bioassays over two generations of wasps. In the first generation, all possible combinations of males and females from the R and S populations were crossed in order to quantify their ability to reproduce on H+ aphids (Table 1). Assuming that this ability is governed by a single Mendelian locus with two alleles (R and S), which are fixed in the respective populations (likely an oversimplification), allowed us to postulate three mutually exclusive hypotheses (H1–H3) that make different predictions for the outcome of these crosses (Table 1). To indicate genotypes and ploidy, crosses are depicted in the following as, e.g., RR × S, meaning that a (diploid) female from the R population was crossed with a (haploid) male from the S population.

**Table 1:** Prediction of female offspring survival and reproduction in experimental crosses of evolved *Lysiphlebus fabarum* populations under three different hypotheses.

Parental genotypes^a^	Offspring genotypes^a^	Female offspring survival on H+ aphids (first generation)			Virgin RS female reproduction on H+ aphids (second generation)	
♀ ♂	♀ ♂	H1: larval dominant	H2: larval recessive	H3: maternal	H3.1: dominant	H3.2: recessive
RR × R	RR R	Y	Y	Y	na	na
RR × S	RS R	Y	N	Y	Y	N
SS × R	RS S	Y	N	N	Y	N
SS × S	SS S	N	N	N	na	na

*R* resistant population, *S* susceptible population, *Y* yes (adult wasps emerged from mummified aphids), *N* no (no adult wasps emerged from mummified aphids), *na* not applicable.

^a^Females are diploid and males are haploid.

(H1) The counteradaptation is larval and dominant. Under H1, RR × R, RR × S, and SS × R crosses are expected to produce female offspring on H+ aphids, as homozygous RR and heterozygous RS female larvae would be of the R phenotype and thus counteradapted. Homozygous SS daughters from SS × S crosses would fail to develop. If the counteradaptation was larval but inherited in an intermediate rather than dominant fashion, the expectation remains the same as under H1, albeit with the possibility that RR × S and SS × R crosses produce fewer female offspring than RR × R crosses.

(H2) The counteradaptation is larval and recessive. Under H2, only RR × R crosses would produce female offspring on H+ aphids. RR × S, SS × R, and SS × S crosses are expected to not produce any female offspring as their heterozygous (RS) or homozygous (SS) daughters would be of the S phenotype and thus not counteradapted.

(H3) The counteradaptation is maternal. Under H3, the RR × R and RR × S crosses are expected to produce female offspring and the SS × R and SS × S crosses are not, as the genotype of the mother is decisive for offspring survival. If both maternal and larval effects were at play, the sex ratio in offspring from the RR×S crosses is expected to be male biased compared to RR × R crosses, due to a disadvantage of RS larvae compared to RR larvae, while haploid male larvae have an R genotype in either case.

To isolate wasps prior to use in experiments, mummies (parasitized aphids approaching parasitoid emergence) were collected and stored individually in 1.5 ml Eppendorf tubes. Thus, adult wasps had never encountered another wasp or aphid before (naive virgins). Zero-to-3 days after hatching, the wasps were paired and given 20–120 min for mating in 1.5 ml Eppendorf tubes. Although there was no control whether mating occurred in the given amount of time, mating was usually observed within the first 30 s of having wasp pairs in the same tube. Then the wasps were released on a caged plant with an aphid colony consisting of a known number of 0–48-h-old H+ aphid nymphs. The mean ± standard deviation (SD) number of aphid nymphs provided per cross was 43.5 ± 14.9. Adult wasps were removed from colonies 24 h after release. Nine days after adding wasps, plants were enclosed in cellophane bags and left to dry out at 22 °C for hatching and subsequent sexing and counting of wasp offspring. Differences in numbers of female offspring between the different crosses of the first generation were analyzed with Mann–Whitney *U* tests. A generalized linear model (GLM) was used to analyze differences in sex ratios. Statistical analyses were performed using R version 3.5.2 (R Core Team 2018).

Because findings from the first generation of crosses supported H3 (see “Results”), two extensions of H3 (H3.1 and H3.2) were tested in a second generation of crosses to determine whether the maternal counteradaptation was dominant or recessive (Table 1). To this end, we tested the ability of 20 virgin female offspring from 10 RR × S crosses (i.e., heterozygous RS females) to reproduce on H+ aphids. The mean ± SD number of aphid nymphs provided per RS female was 21.9 ± 9.5.

(H3.1) The counteradaptation is maternal and dominant. Under H3.1, RS females are expected to reproduce successfully on H+ aphids, because they are of the R phenotype. They are expected to produce only male offspring as they are virgins (arrhenotokous parthenogenesis). This scenario is indistinguishable from cytoplasmic inheritance, which would require further examination.

(H3.2) The counteradaptation is maternal and recessive. Under H3.2, RS females are not expected to reproduce on H+ aphids, because they are of the S phenotype.

### Experiment 2: crosses and phenotyping for QTL study

**Fig. 1:**
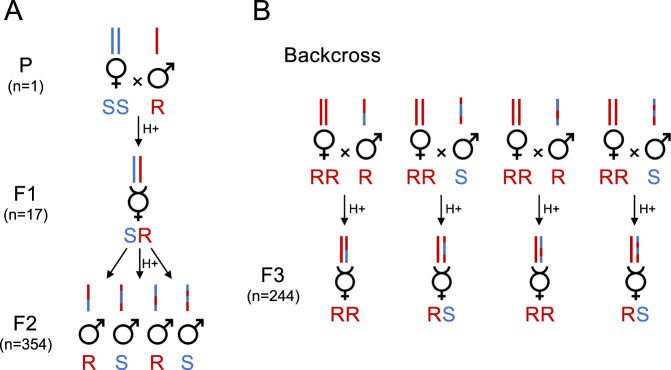
Experimental crossing procedure for QTL analysis. Crossing design used to obtain a F2 mapping population for genotyping (**A**) and a F3 population for phenotyping (**B**). In a first step, two P generation individuals (parents), a diploid female from the symbiont-susceptible population, and a haploid male from the symbiont-resistant population were crossed to obtain 17 heterozygous F1 hybrid females. F1 hybrid females were allowed to reproduce as virgins—i.e., arrhenotokous parthenogenesis—to obtain 354 recombinant F2 males (mapping population), which were either carrying the S (susceptible) or the R (resistant) genotype. Recombinant F2 males were backcrossed with females of the resistant population to produce semi-recombinant F3 females. Sister F3 females have identical chromosomes of paternal origin and are thus considered clonal sibships. Two hundred and forty-four clonal sibships consisting of one to two sister F3 females were allowed to reproduce as virgins on a colony of symbiont-protected (H+) aphid hosts for phenotyping. Bar colors represent genomic regions originating from different parental populations and letters under sex symbols indicate the ploidy levels and genotypes.

To obtain a mapping population and phenotype data, a crossing scheme similar to the one by Pannebakker et al. (2011) was realized (Fig. 1). The crossing design relied on two main assumptions: First, we assumed that the alleles responsible for the counteradaptation are fixed in alternative states in the R and S populations. Second, due to the findings from the first experiment, we assumed the counteradaptation to be recessive and determined by the maternal genotype (see “Results”). In the first generation (P generation), a single S female was crossed with an R male to produce heterozygous female RS offspring (F1 generation). F1 females were allowed to reproduce as naive virgins to produce a recombinant male-only mapping population (F2 generation, Fig. 1A). F2 males were then backcrossed into the R background (each male with one RR female) to produce F3 female offspring for phenotyping (Fig. 1B). All reproduction up to the emergence of F3 females took place on H− aphids (Fig. 1) to avoid any selection. P individuals, F1 females and F2 males were stored in 1.5 ml Eppendorf tubes at −80 °C for subsequent genotyping.

Phenotyping was conducted by letting naive virgin F3 females oviposit for 24 h on colonies with a known number of approximately 24–72-h-old H+ aphid nymphs and subsequently counting their offspring as previously described. The average ± SD number of aphid nymphs provided was 40.9 ± 13.6. Wasps were added to the aphid colonies in an open Eppendorf tube. If possible, two sister F3 females from the same recombinant F2 father were added to each aphid colony in order to reduce the occurrence of false negatives, i.e., random failures to reproduce that are unrelated to the females’ genotype, e.g., due to harmful handling or death before oviposition. F3 sister females are identical concerning their paternal chromosome set and share the same R population background concerning their maternal chromosome set. They are considered clonal sibships (Pannebakker et al. 2011).

The phenotype we measured was the number of wasp offspring produced per H+ aphid colony. This measure exhibited strong variation and zero inflation. To improve its value as a proxy for counteradaptation, the measure was corrected for certain variables in the phenotyping set-up that could have influenced offspring production independent of the F3 genotype. We used the zeroinfl function of the R-package pscl (Zeileis et al. 2008) to fit the following full model by zero-inflated Poisson regression:

*n_offspring* ~ *n_nymphs* + *n_wasps_added* + *all_removed* + *any_found_dead* + *any_in_tube* | *n_nymphs* + *n_wasps_added* + *all_removed* + *any_found_dead* + *any_in_tube*

where *n_offspring* is the number of offspring wasps produced, *n_nymphs* is the number of aphid nymphs, i.e., potential hosts, provided, *n_wasps_added* is a factor describing whether one or two wasps were added to the aphid colony, *all_removed* is a factor describing whether all wasps could be recovered 24 h after adding them to the aphid colony, *any_found_dead* is a factor describing whether any of the wasps were dead after 24 h, and *any_in_tube* is a factor describing whether any of the wasps were found in the tube rather than on the plant after 24 h. Parameters before and after the | symbol are components of the Poisson and the zero-inflation part of the model, respectively. The full model was reduced to a minimal model by performing backwards elimination with the function be.zerofinl from the R-package mpath (Wang 2020). The final minimal model was:

*n_offspring* ~ *n_nymphs* + *all_removed* + *any_in_tube* | *n_wasps_added* + *any_in_tube*.

Residuals of the minimal model were used as the corrected count phenotype for QTL mapping. We also assessed offspring presence presence/absence as an additional binary phenotype. Due to its simplicity, a binary phenotype may be less prone to environmental variation and more appropriate if counteradaptation is a Mendelian trait.

### DNA extraction and sequencing

DNA extraction from 354 F2 males, 17 F1 females, and the two P individuals was performed adapting the LGC-sbeadex Livestock D protocol (LGC Genomics, Berlin, Germany). In addition to these experimental individuals, 30 wasps from an asexual, isofemale line of *L. fabarum* (line CV17-84) were processed to quantify genotyping error. Due to their mode of reproduction and maintenance at small population size, CV17-84 individuals are expected to be genetically nearly identical. F2, F1, and P individuals and three pools of 10 CV17-84 wasps each were crushed in liquid nitrogen prior to lysis. Extraction from individual samples was downscaled and included the following adaptations: lysis was done with PN buffer during 2 h at 60 °C with 1:10 protease solution, the lysate was incubated with binding mix during 20 min and elution was done at 60 °C. Extraction from pooled samples was, besides doubling the amount of protease, done following the manual. DNA concentration of each sample was measured using a Spark 10 M Multimode Microplate Reader (Tecan, Switzerland). Quality of DNA obtained with the used protocols was tested on a Nanodrop spectrophotometer (Thermo Fisher Scientific, USA) and on agarose gels. ddRAD library preparation was adapted from the protocol by Peterson et al. (2012). Restriction enzymes MfeI and TaqI were used for double digestion of up to 50 ng DNA per sample. After ligation of barcoded adapters to each individual sample, samples were combined in 12 pools with 24–36 samples each. Eleven pools contained one sample of 50 ng DNA from the CV17-84 wasps and 23–35 other samples (F2, F1, or P). Fragment size selection was performed on each pool with AMPure XP beads (Beckman Coulter, USA) (0.6× and 0.09×) and followed by selection of biotinylated P2 adapters. This was followed by PCR with KAPA HiFi HotStart ReadyMix (Roche, Switzerland) to amplify DNA and add 12 different Illumina primers to identify pools. Pools were then purified and combined into a final library. Mean fragment size of the library was 606 bp, as measured with the 2200 TapeStation (Agilent, USA), which corresponds to a mean insert size of 470 bp. The library was sequenced in a single lane of an SP flow cell on an Illumina NovaSeq 6000 System with 2 × 150 bp paired-end sequencing (at Functional Genomic Center, Zürich). A total of 307.2 million paired-end reads were obtained from P, F1, and F2 individuals and 11 CV17-84 control samples.

### Genotyping

We used the dDocent pipeline (Puritz et al. 2014; Puritz et al. 2014) for genotyping. Reads were demultiplexed with the *process_radtags* function of the STACKS package (v 2.14, Catchen et al. 2013) with disabled filtering of degraded cut sites, which led to 304.2 million demultiplexed paired-end reads. BWA-MEM (v 0.7.17, Li and Durbin 2010) was used with default settings to map reads to the reference genome of *L. fabarum* (Lf_genome_V1.0.fa, Dennis et al. 2020). On average (±SD), 1.585 (±1.058) million reads were assigned per sample during demultiplexing. out of which an average of 81.66% were mapped and retained after filtering for mapping quality (Supplementary Table S1). We called 547,092 variants using freebayes (v 1.3.1, Garrison and Marth 2012) with the default settings from dDocent pipeline specifying population (corresponding to the generation P, F1, F2, or CV17-84) and ploidy of individuals. The VCF-file was then split into a dataset containing 355 haploid individuals, i.e., males (one P, 354 F2) and a dataset with 29 diploid individuals i.e., females (1 P, 11 CV17-84, 17 F1). The dataset with diploids was filtered following the dDocent filtering pipeline up until removing indels, retaining 2456 single-nucleotide polymorphisms (SNPs). The following changes were made to the tutorial: the minimum quality score (–*minQ*) was set to 20, the minimum mean depth (–*min-meanDP*) was set to 10, and the maximum mean depth (–*max-meanDP*) was set to 400. The haploid dataset was then transformed to allelic primitives and filtered to contain only the 2456 SNPs that were retained in the diploid dataset. The VCF files containing haploid and diploid samples were then transformed to SNP tables using samtools (v 1.9, Li et al. 2009) and custom bash scripts. A custom R-script was then used to filter the SNP tables and create an input file for linkage mapping with MSTmap (Wu et al. 2008). The retained SNPs are homozygous in the mother, biallelic among the two parent individuals, and known in both parent individuals. Additionally, we tested for segregation distortion, removing SNPs that deviate significantly from an allele frequency of 50% based on a chi-square test with Bonferroni-corrected false-discovery rate of 5%. For each allele in each offspring (F2) male, alleles were recoded as “A” for maternal, “B” for paternal, and “U” for unknown. SNPs missing in >50% of individuals and individuals with >50% unknown genotypes were removed. The dataset used for linkage mapping contained 351 F2 individuals and 1838 SNPs of which 3 were removed by MSTmap internal filters leading to a final dataset of 1835 SNPs contained in the linkage map.

### Quantification of genotyping error

Genotyping error rate was quantified by counting mismatches between the supposedly identical genotypes of 11 CV17-84 DNA samples that were sequenced as part of 11 different pools. The 1835 SNPs used for QTL mapping were used as a template to filter SNPs in the dataset with CV17-84 individuals with vcftools (–*positions* flag). A SNP table containing CV17-84 genotypes was then analyzed in R to quantify genotyping error. For each pair of CV17-84 samples, the proportion of genotype mismatches was counted and averaged over all comparisons to obtain an estimate of mean genotyping error. Unknown genotypes were not counted as mismatch. The mean percentage of pairwise mismatches among the 11 CV17-84 samples ranged from 0.8392 to 1.706% with an average of 1.207%. The average mismatch measure was employed as an estimate for the genotyping error during analyses with R/qtl (Broman et al. 2003).

### Linkage map and QTL mapping

Linkage mapping was performed with MSTmap (Wu et al. 2008) using the following settings: *population_type* = *DH, distance_function* = *kosambi, cut_off_p_value* = *0.000001, no_map_dist* = *15.0, no_map_size* = *2, missing_threshold* = *0.25, estimation_before_clustering* = *no, detect_bad_data* = *yes, objective_function* = *COUNT*. The resulting distance matrix was processed with R to contain only marker locations, Linkage group (LG) ID, and map distance. The new linkage map was edited in order to use the same LG IDs and orientations as in the linkage map by Dennis et al. (2020).

Phenotype data, genotype data, and the new linkage map were merged with a custom R script to produce an input file for R/qtl (Broman et al. 2003). After reading the dataset with R/qtl, its cross type was transformed to recombinant-inbred by selfing (*convert2riself* function) because this expects no heterozygotes and genotype frequencies at 0.5, which fits our crossing scheme. We tested for duplicated genotypes (>90% similarity between individuals), checked for switched markers using the *checkAlleles* function, and plotted recombination fractions (Supplementary Fig. S1), none of which indicated any problems. Intermarker distance was estimated with the *est.map* function, setting map function to “kosambi” and tolerance to 10^−4^. The resulting map was used as new linkage map with cM as map unit. Conditional genotype probabilities were calculated at a step size of 0.1 cM. The *scanone* function was used to calculate logarithmic of the odds (LOD) scores over the genome using the default (EM) algorithm with nonparametric and binary model for the corrected count phenotype and the additional binary phenotype, respectively. Significance thresholds were calculated by conducting 1000 permutations and choosing a 5% cut-off corresponding to the significance threshold at an alpha of 5%. The 95% approximate Bayes confidence interval was then calculated for the chromosome with significant LOD score. After simulating genotypes 1000 times with a step size of 0.1 cM and pulling genotype probabilities at the peak LOD, the explained phenotypic variance was estimated with the *fitqtl* function.

### Candidate gene identification

As RADseq loci are usually short and represent a small proportion of the genome, they are unlikely located in candidate genes themselves. The 95% approximate Bayes confidence interval of the single significant QTL we identified includes all markers on scaffold tig00000002, upwards of 311,170 (bp). Thus, we considered tig00000002 from position 311,170 on as region for searching candidate genes. Gene annotations were retrieved from the recently published *L. fabarum* genome (Dennis et al. 2020). In addition, we identified putative venom and toxin genes in the *L. fabarum* genome in order to explore this function among candidate genes. To do so, we collected venom protein sequences from several parasitoid wasp species: *Nasonia vitripennis* (Danneels et al. 2010), *Chelonus inanitus* (Vincent et al. 2010), *Microplitis demolitor* (Burke and Strand 2014), *Fopius arisanus* (Geib et al. 2017), *Diachasma alloeum* (Tvedte et al. 2019), *Cotesia congregata* (Gauthier et al. 2021), *Leptopilina boulardi*, *Leptopilina heterotoma* (Goecks et al. 2013), and *Aphidius ervi* (Colinet et al. 2014); and retrieved candidate animal toxin proteins (7151 sequences) from the UniProt Animal Toxin Annotation Program database (UATdb, Jungo et al. 2012). These proteins were then matched to *L. fabarum* proteins by blastp (*-e-value* < *1e-8*, *-max_target_seqs* = *10*, Camacho et al. 2009). This was combined with the 32 *L. fabarum* proteins identified as venoms by proteomic studies (Dennis et al. 2020).

## Results

### Nature of the counteradaptation

**Fig. 2:**
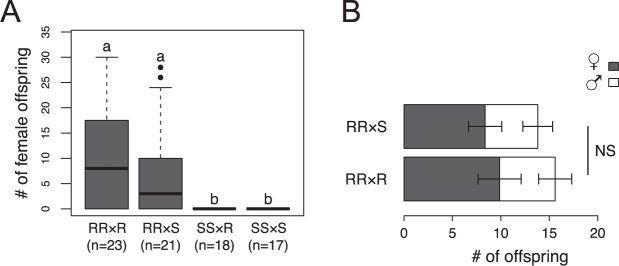
Reproduction of crossed *Lysiphlebus fabarum* populations on *Hamiltonella*-protected aphids. **A** Female offspring numbers produced by the four different crosses between resistant (R) and susceptible (S) *L. fabarum* wasps on H+ aphids. Numbers of offspring correspond to the reproduction of a male and a female wasp that were given 24 h to reproduce on a H+ aphid colony. Letters indicate significant differences assessed by Mann–Whitney *U* tests (RR × R vs RR × S: *U* = 200, n1 = 23, n2 = 21, *P* = 0.32; RR × R vs SS × R: *U* = 59.5, n1 = 23, n2 = 18, *P* < 0.001; RR × R vs SS × S: *U* = 63, n1 = 23, n2 = 17, *P* < 0.001; RR × S vs SS × R: *U* = 68, n1 = 21, n2 = 18, *P* < 0.001; RR × S vs SS × S: *U* = 72, n1 = 21, n2 = 17, *P* < 0.001). **B** Comparison of offspring sex ratio between the two successful crosses. The stacked plot represent mean ± SEM number of offspring individuals emerging per cross. GLM with quasibinomial error distribution and dispersion parameter taken to be 3.607 revealed no significant (NS) difference between sex ratios.

To determine the nature of the counteradaptation of *L. fabarum* to *Hamiltonella*-protected aphids, wasps from the R and S populations were crossed prior to assessing reproduction on H+ aphids (Table 1). Out of 23 RR × R, 21 RR × S, 17 SS × R, and 18 SS × S crosses, female offspring were observed exclusively in 16 RR × R and 13 RR × S crosses (Table 2 and Fig. 2). Mann–Whitney *U* tests (followed by Bonferroni correction of *P* values) showed significantly different numbers of female offspring in all four comparisons of crosses with RR mothers against crosses with SS mothers (*P* < 0.001) but not when comparing RR × R against RR × S crosses (*P* = 0.32, Fig. 2a). The fact that female offspring are only produced in crosses with R mothers is in line with H3, suggesting that counteradaptation is governed by the maternal genotype (Table 1). H1 and H2 can be ruled out because both predict equal numbers of female offspring in RR × S and SS × R crosses (Table 1). A GLM test showed that sex ratios did not differ between RR × S and RR × R crosses (Fig. 2b, *P* = 0.730). Therefore, no evidence was found for additional larval effects that would lead to differences in sex ratios between these crosses. In the second generation, 20 virgin (RS) female descendants from 10 RR × S crosses were allowed to oviposit on H+ aphids (Table 1). None of these RS females produced any offspring. This supports H3.2 and indicates that the counteradaptation is maternal and recessive. Additionally, it excludes that a cytoplasmic element is responsible, because the RS females had inherited the cytoplasm from their H+-adapted RR mothers.

**Table 2:** Observed offspring numbers from experimentally crossed resistant (R) and susceptible (S) *Lysiphlebus fabarum* populations on *Hamiltonella*-protected aphid host.

Cross	Crosses with reproduction^a^	Female offspring			Male offspring		
		Mean	SEM	Median	Mean	SEM	Median
RR × R	16/23	9.87	1.98	8	5.74	1.71	5
RR × S	13/27	8.38	2.22	3	5.43	1.53	4
SS × R	0/17	0	0	0	0	0	0
SS × S	0/18	0	0	0	0	0	0

^a^For each cross, a wasp pair consisting of a male and a female was given 24 h for reproduction and oviposition on an aphid colony.

### Genotyping and linkage map construction

**Fig. 3:**
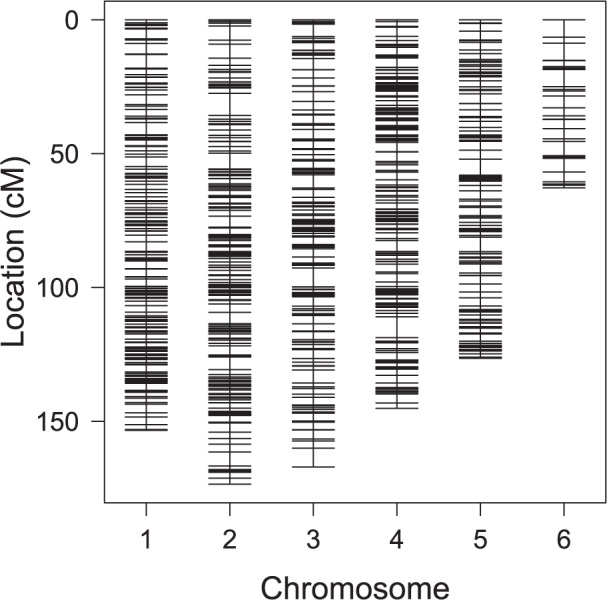
Linkage map of the *Lysiphlebus fabarum* F2 cross. Repartition of the 1835 high-quality SNP markers over the six inferred linkage groups (LGs) representing a total size of 828.3 cM.

From our backcross scheme (Fig. 1) starting with a single P generation cross, we obtained 17 F1 individuals that produced 354 F2 males (mapping population). The dataset used for linkage mapping and QTL analyses contained 351 F2 males and 1835 SNPs. The linkage map created from this dataset contained six LGs (Fig. 3), likely corresponding to the six chromosomes identified in *L. fabarum* by karyotyping (Belshaw and Quicke 2003). Estimation of intermarker distance with R/qtl yielded a total map size of 828.3 cM with an average marker spacing of 0.4529 cM and a maximum distance between markers on the same LG of 8.265 cM (Table 3 and Supplementary Table S2). The 1835 SNPs were located across 352 scaffolds, representing 20.73% of the 1698 scaffolds from the *L. fabarum* reference genome assembly. The scaffolds included in the linkage map account for 59.94% (84.34 Mbp) of the 140.7 Mbp *L. fabarum* reference genome. Eight scaffolds were found to map on two different LGs (Supplementary Table S2) and are thus likely chimeric. Given the genome size and map length, the recombination rate of *L. fabarum* was estimated to be 5.887 cM/Mbp. This recombination rate and the high marker density were both favorable to QTL detection. The linkage map resembles another recently constructed in terms of LG number, size, and included scaffolds (Matthey-Doret et al. 2019, Dennis et al. 2020). However, our map provides a slight improvement compared to the previous one in Dennis et al. (2020), which contained approximately 53% of the reference genome of *L. fabarum* (almost 60% for ours).

**Table 3:** Genetic map summary.

Linkage group	Number of markers	Length (cM)	Length (Mbp)	Average spacing (cM)	Maximum spacing (cM)
1	368	153.354	17.5	0.417	5.121
2	377	173.508	18.6	0.461	8.265
3	304	167.050	15.3	0.551	7.035
4	421	145.142	14.7	0.345	7.737
5	304	126.455	13.6	0.417	5.965
6	61	62.811	4.4	1.046	6.483
Overall	1835	828.322	84.3	0.452	8.265

Length (Mbp) represents the total length of scaffolds included in the linkage group/map.

### Main QTL for counteradaptation

By backcrossing F2 males with R population females, we obtained 244 F3 sibships for phenotyping. F3 females were tested for their ability to produce offspring on H+ aphids. Three sibships were excluded due to missing F2 genotypes. Offspring were observed in 153 out of 241 sibships, which was significantly >50% of the sibships (*χ*^2^ = 17.53, df = 1, *P* < 0.001). The average number of offspring per sibship (±SEM) was 5.988 (±0.4472), across the range of 0–28. The binomial part of the zero-inflation model showed that the probability to observe offspring in phenotyping colonies was affected by two variables. It was increased when two, instead of one, F3 females were added (*n_wasps_added* variable) and when both wasps had left the tubes within which they were added to the colony (*any_in_tube* variable). Offspring counts generally increased with aphid colony size (Supplementary Fig. S2 and Supplementary Table S3). The residuals from this model were taken as corrected count phenotypes for QTL mapping. We additionally used the binary variable of offspring presence/absence as phenotype.

**Fig. 4:**
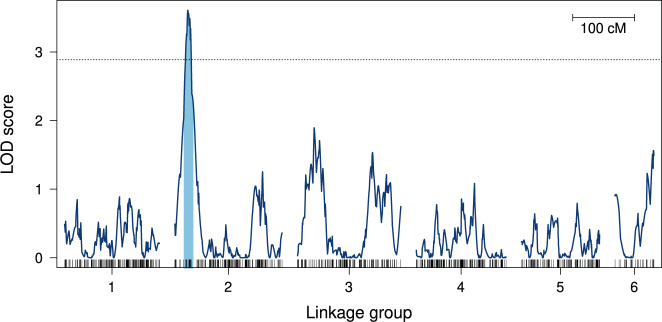
QTL mapping. LOD scores of the nonparametric QTL model reveal a significant peak in LG 2. LOD scores were calculated at marker locations and between markers at 0.1 cM intervals. The analysis was carried out with a corrected count phenotype representing counteradaptation. The horizontal dotted line shows the global significance threshold for *P* = 0.05, determined by permutation test (*N* replicates = 1000). The blue region on LG 2 represents the 95% approximate Bayes confidence interval for the QTL located on this linkage group. Vertical ticks at the bottom show marker locations for each linkage group.

Two hundred and forty-one phenotype observations and 1835 SNPs were used for the QTL analysis with corrected count phenotypes. The significance threshold at an alpha of 5% was found to be at a LOD of 2.887. A single genomic region exhibited a LOD score above the significance threshold (Fig. 4). The peak LOD score of 3.608 occurred on LG 2 at 21 cM (*P* = 0.008). The marker with the maximum LOD score of 3.517 was located nearby at 21.72 cM. The region with LOD score above the significance threshold on LG 2 ranges from 17.2 to 26.5 cM. The 95% approximate Bayes confidence interval ranges from 15.1 to 29.3 cM. The single-QTL model could explain 7.25% of the observed phenotypic variation (Supplementary Fig. S3). Using the additional binary phenotype, a maximum LOD score of 1.733 (*P* = 0.544) was observed at 20.6 cM on LG 2, but no significant QTL was found (Supplementary Fig. S4).

### Candidate genes

The QTL for counteradaptation to *H. defensa* identified on the LG 2 maps to positions on scaffold tig00000002 upwards of 311,170 bp of the *L. fabarum* reference genome. This region corresponds to approximately 1.17 Mbp of the scaffold tig00000002. According to the genome annotation, 120 coding genes are included in this region (Supplementary Table S4) and are thus candidate genes for the counteradaptation. Among these genes, nine genes may be of particular interest as they encode homolog proteins to venom proteins from other parasitoid wasp species or animal toxins (Table 4).

**Table 4:** Putative venom genes among the 120 candidate genes for counteradaptation to *Hamiltonella* located in the 95% Bayes confidence interval of the QTL identified in LG 2.

Gene ID	Candidate category^a^	Annotated function	Distance from max. LOD score marker (Kbp)^b^
LF000355	Toxin	Seminal metalloprotease 1	147.77
LF000292	Venom	Protein yellow	708.93
LF000293	Venom	Chymotrypsin-1-like	700.24
LF000294	Venom	Thioredoxin	698.47
LF000308	Venom	Plasma membrane calcium-transporting ATPase 2	487.21
LF000319	Venom	Neprilysin-like	324.01
LF000324	Venom	Rho GAP 44-like	257.17
LF000401	Venom	Eukaryotic peptide chain release factor GTP-binding subunit 3A	418.39
LF000408	Venom^c^	Venom low-density lipoprotein receptor-related protein	444.03

^a^Venom and toxin: genes encoding for proteins matching parasitoid wasp venoms or animal toxins (determined by blastP).

^b^The middle point of each gene was considered to calculate the distance from the maximum LOD score marker.

^c^Venom protein identified by mass spectrometric analysis of venom glands (Dennis et al. 2020).

## Discussion

This study represents the first attempt to investigate the genomic basis of the counteradaptation to symbiont-conferred resistance in aphid parasitoids with a QTL approach. We found that counteradaptation is a recessive trait and determined by the maternal genotype. Further, we identified a QTL region that is associated with the counteradaptation. This was possible because we had at our disposal wasp lines diverging in their ability to parasitize symbiont-protected aphids from a previous experimental evolution study (Dennis et al. 2017).

Although we detected a single significant QTL associated with the counteradaptation to *Hamiltonella*-protected aphids on *L. fabarum*’s LG 2, which would in principle be consistent with single-locus Mendelian inheritance, the genetic determination of the counteradaptation is unlikely to be that simple. With a single contributing locus, about 50% instead of the observed 36.51% of F3 sibships should have been unable to reproduce on H+ aphids, likely leading to a clearer separation of phenotypes by the genotype (Supplementary Fig. S3), as it is the case, for example, for an insecticide-resistance QTL in bed bugs (Fountain et al. 2016). Moreover, a single-locus inheritance of the counteradaptation would likely have been detected in the additional analysis with offspring presence as binary phenotype, which detected no significant QTLs (Supplementary Fig. S4). The count-based phenotype may be more powerful at capturing loci associated with producing large numbers of offspring on H+ aphids. Thus, the identified QTL is likely associated with the success rate of parasitizing H+ aphids. Furthermore, the identified QTL may not represent the only region contributing to counteradaptation. With the count-based phenotype, we do indeed see other genomic regions with elevated LOD scores, e.g., on LG 3 (Fig. 4), but these were not statistically significant. Finally, because we did not find any evidence for a contribution of the offspring genotype in experiment 1, our breeding design was not optimized to examine larval contributions acting in addition to the maternal counteradaptation we screened for. The existence of a larval contribution can thus not be ruled out.

We estimated that the QTL on LG 2 explains around 7.25% of the phenotypic variance in reproductive success on H+ aphids. This percentage may seem low, but other QTL analyses in parasitoid wasps have also identified loci making modest contributions to the phenotypic variance. For example, QTLs identified by Pannebakker et al. (2011) explained 0.31 and 0.16% of the phenotypic variance in clutch size and sex ratio in *N. vitripennis*, respectively. Using a crossing design that involved the two species *N. vitripennis* and *N. giraulti*, Werren et al. (2016) found a QTL that explained 14% of the phenotypic variance in head width-to-head length ratio. In *C. congregata*, even 27.7 and 24.5% of the phenotypic variance observed in parasitism success and offspring number could be explained by individual QTLs (Benoist et al. 2020). It is possible that we underestimated the importance of the counteradaptation QTL, despite the size of our mapping population being close to the suggested range of 250–300 for QTL studies in *N. vitripennis* (Gadau et al. 2012), because the environmental variance (*V*_E_) was certainly high. We observed a lot of variation in reproduction and even the presence of non-reproductive individuals from the pure R population in the first crossing experiment. This indicates that reproduction on H+ aphids is a trait that is difficult to measure precisely, and the absence of reproduction on *Hamiltonella*-protected aphids does not necessarily reflect an absence of the ability to do so. We tried to reduce this variation somewhat by using the residuals of a zero-inflated GLM that corrected for some sources of *V*_E_, but the effect was limited. A possible improvement would consist in increasing the number of phenotyped sister F3 females to obtain a more accurate measure of their phenotype (Benoist et al. 2020). Additionally, a better control of the experimental set-up to reduce *V*_E_ (e.g., equal numbers of aphids available to parasitize) could contribute to a more precise measure of the counteradaptation trait.

Our observation that the counteradaptation of *L. fabarum* to the *H. defensa* strain H76 is mainly determined by the maternal genotype is somewhat surprising, given that the larvae have to complete their development in a host that contains toxin-producing symbionts, and it suggests that the relevant effectors are deposited at oviposition. They may comprise maternal contributions to the egg or belong to the manifold compounds females inject alongside the egg to suppress host immunity and thereby prime hosts for successful development of their offspring (Schmidt et al. 2001). We can thus speculate that the molecules injected by the wasp with its eggs may impair the protection conferred by *Hamiltonella* and its associated APSE phage, although it is still unknown if the counteradaptation indeed targets the bacteria directly or rather increases offspring tolerance of these bacteria. An important maternal mechanism making up the parasitism arsenal of many parasitoid wasps are symbiotic polydnavirus particles, virus-like particles, and venoms (Burke and Strand 2014). As the genome of *L. fabarum* is not known for having integrated a polydnavirus (Dennis et al. 2020), unlike other wasps from the Braconidae family (Gauthier et al. 2018), venoms are likely involved in the counteradaptation, as also suggested by transcriptome data in Dennis et al. (2017). The venoms of parasitoid wasps can serve various purposes as they induce a variety of physiological and behavioral changes in the host (Moreau and Asgari 2015). Even antimicrobial properties were shown in the case of the cysteine-rich peptide defensin-NV from *N. vitripennis* venom (Ye et al. 2010). Moreover, genes encoding wasp venom are rapidly evolving and diverse both among and within parasitoid wasp species (Cavigliasso et al. 2019), making them suitable targets for rapid evolution in an arms race between parasitoids and host-protective endosymbionts (Colinet et al. 2010). In the identified QTL candidate region, 9 genes out of 120 encode *L. fabarum* putative venoms, but they are not in the close vicinity of the peak with maximum LOD score (Supplementary Table 4). Indeed, among the closest candidate genes, none are predicted as putative venom genes. However, since the blast approach we used here identifies only parasitoid conserved venoms and the proteomic approach generally identifying a limited number of proteins (Dennis et al. 2020), we cannot exclude that some of the unknown proteins encoded by the candidate genes are *L. fabarum* specific venoms. Further tissue-specific analyses of venom glands would be needed to have an exhaustive overview of *L. fabarum* venoms.

The ability to parasitize *Hamiltonella*-protected aphids being a recessive trait—at least in the case of *Hamiltonella* strain H76—has implications for the evolution of parasitoid counteradaptation. Recessive alleles persist in a population for a long time even if the trait they determine is no longer under positive selection or becomes selected against (Agrawal and Whitlock 2012). Previous studies on this system found no obvious costs of parasitoid counteradaptation, but it was highly specific to the symbiont strain the parasitoids were confronted with (Rouchet and Vorburger 2014; Dennis et al. 2017). In a dynamic system with some turnover of symbiont strains, e.g., if fueled by negative frequency-dependent selection (Agrawal and Lively 2002; Kwiatkowski et al. 2012), prior adaptations may no longer be useful if a new symbiont strain becomes prevalent in the host population. They could even be detrimental if counteradaptations to different strains were mutually exclusive (currently unknown). Such turnover would, however, leave a legacy of prior adaptations in the form of “invisible” recessive alleles in the heterozygous state within the parasitoid population. They would form the substrate for adaptation from standing genetic variation (Barrett and Schluter 2008), ready to respond to future selection from defensive symbionts. This scenario is certainly consistent with the rapid speed of parasitoid counteradaptation observed under experimental evolution (Dion et al. 2011; Rouchet and Vorburger 2014; Dennis et al. 2017).

Combining crossing experiments with a QTL analysis, we showed that the counteradaptation of *L. fabarum* to *Hamiltonella*-protected aphids is a maternally determined trait, and we identified an associated QTL. This QTL explained only a low proportion of the phenotypic variance in the wasps’ ability to parasitize H+ aphids, a difficult-to-measure trait that appears to suffer from a lot of environmental noise. The genomic region covered by this QTL contains several putative venom genes, potential candidates for targeting *H. defensa* and its bacteriophage, which are responsible for parasitoid resistance in aphids. Improvements in phenotyping and further studies (e.g., genome wide approaches) will be necessary to explain a larger proportion of the variance in wasp parasitism success on protected aphids and to narrow in on the responsible genes.

It would be premature to draw any general conclusions for the evolution of parasite counterdefenses against symbiont-mediated protection from our study, and the literature does not offer many other examples to compare it with. General conclusions may also be difficult for a more fundamental reason: the counteradaptations required for parasites to overcome symbiont-conferred resistance will depend on the mechanism by which the resistance is achieved. These mechanisms differ widely among defensive symbionts (reviewed in Gerardo and Parker 2014). One interesting example pertains to our very study system. Black bean aphids are also protected against *Lysiphlebus* parasitoids by tending ants, which can be regarded as macrobial protective symbionts (Stadler and Dixon 2005). The parasitoids’ counteradaptation against these symbionts consists in the evolution of chemical mimicry. The cuticular hydrocarbons of *Lysiphlebus* wasps mimic those of their aphid hosts, preventing their removal from aphid colonies by ants (Liepert and Dettner 1996).

For bacterial defensive symbionts, one generality that has emerged is that host protection is often mediated by toxins (Ford and King 2016; Oliver and Perlman 2020), but the types of toxins may differ among symbiont strains, as shown for *H. defensa* (Degnan and Moran 2008; Oliver and Higashi 2019). In the experimental evolution study by Dennis et al. (2017), wasps were also evolved in the presence of a second strain of *H. defensa* (H402, carrying another APSE variant that encodes a different toxin), equally resulting in rapid and specific counteradaptation. The H402-adapted wasps were not included in the present study, but it would be interesting to compare the genomic basis underlying their counteradaptation to see whether the same or different genomic regions are involved. The involvement of different regions would support an idiosyncratic nature of parasitoid counteradaptation in response to particular defense mechanisms, and it could help to explain the observed specificity of *H. defensa*–*L. fabarum* interactions.

## Supplementary information

Supplementary figures

Supplementary table S1

Supplementary table S2

Supplementary table S3

Supplementary table S4

## Data Availability

Sequencing data related to this study can be found under the accession PRJEB39724 in the European Nucleotide Archive (https://www.ebi.ac.uk/ena/browser/home). Count data, phenotypes, genotypes, and scripts used to perform the different analyses of this study are available at 10.5061/dryad.18931zcvz.
