# Supplementary material for: Quantitative trait locus analysis of parasitoid counteradaptation to symbiont-conferred resistance
Source: Heredity (Edinb). 2021 May 19;127(2):219–32. doi: 10.1038/s41437-021-00444-7 (PMC8322320; doi:10.1038/s41437-021-00444-7)
Supplement: Supplementary file 1 — Supplementary figures [file 41437_2021_444_MOESM1_ESM.pdf]

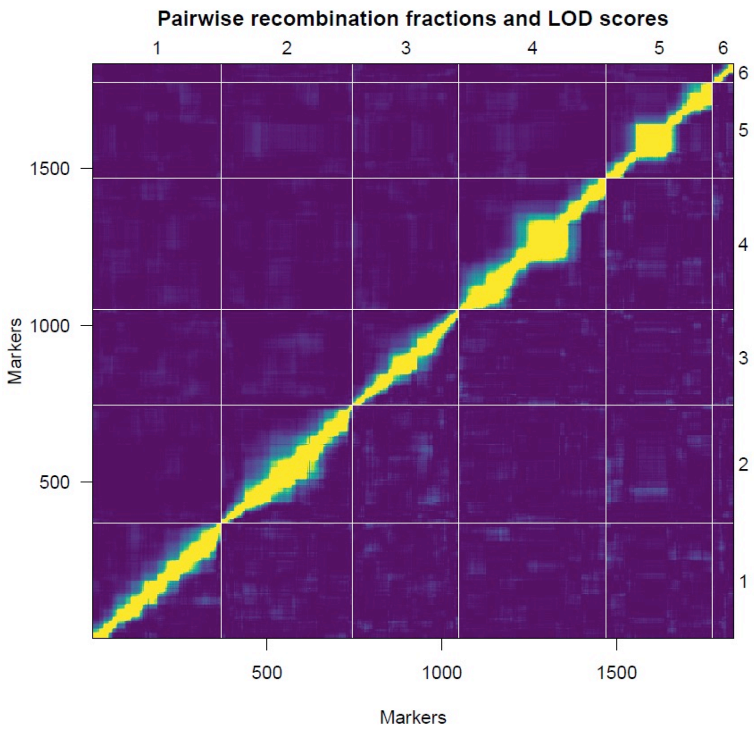

Figure S1. Pairwise recombination fractions and LOD scores of the linkage map with 1835 SNPs. This diagnostic plot was used to assess linkage map quality and was made with the R/qtl command *plot.rf*. Pairwise estimated recombination fractions r are shown in the upper left. LOD scores for the test $r = 0.5$ are shown in the bottom right. Yellow indicates linked markers (low r or high LOD) and blue indicates unlinked markers (high r or low LOD).

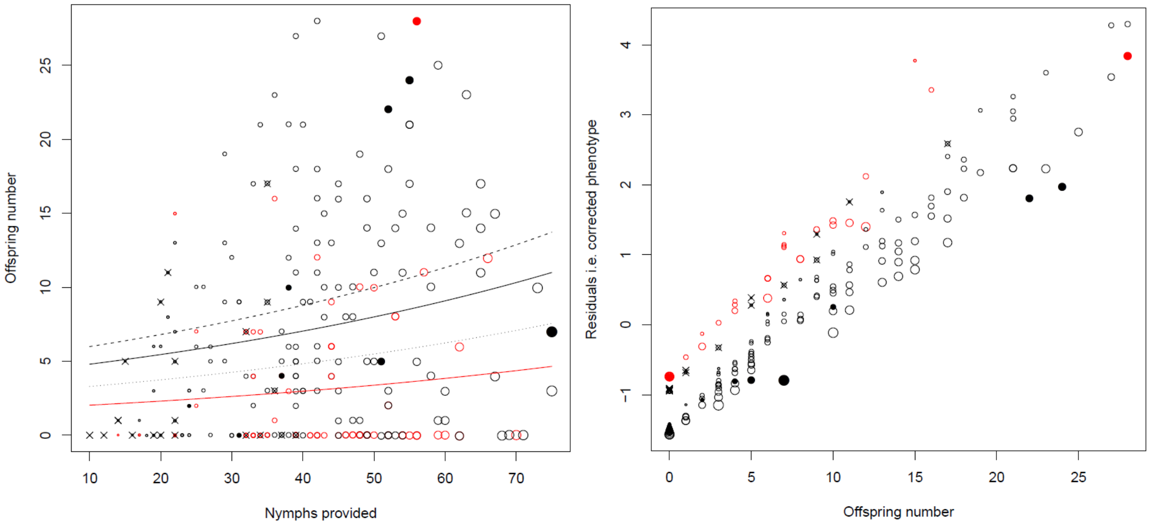

Figure S2. Model fit explaining variation in the count phenotype with zero-inflated Poisson regression to obtain phenotype values that are corrected for variables in the experimental setup. Point size is proportional to the number of aphid nymphs provided. Point color indicates whether some (red) or no (black) wasps were found still in the tube upon removal of wasps. Finding wasps in the tube may indicate absence of oviposition behavior which could lead to lower reproduction. Filled points indicate that only one wasp was removed instead of two, increasing the offspring number. Overlaid crosses indicate that only one wasp was added at the beginning of the experiment, reducing the number of offspring. **Left panel:** offspring numbers vs nymphs (potential hosts) provided. Lines show the predicted offspring number for different combinations of factorial variables. The solid black line is the prediction for open black points (all removed, none in tube, two added). The solid red line is the prediction for open red points (all removed, some in tube, two added). The dashed black line is the prediction for solid black points (not all removed, none in tube, two added). The dotted line is the prediction for open black points with an overlayed cross (all removed, none in tube, one wasp added). **Right panel:** Relationship of offspring number and corrected phenotype values (i.e. residuals of the zero-inflated Poisson regression).

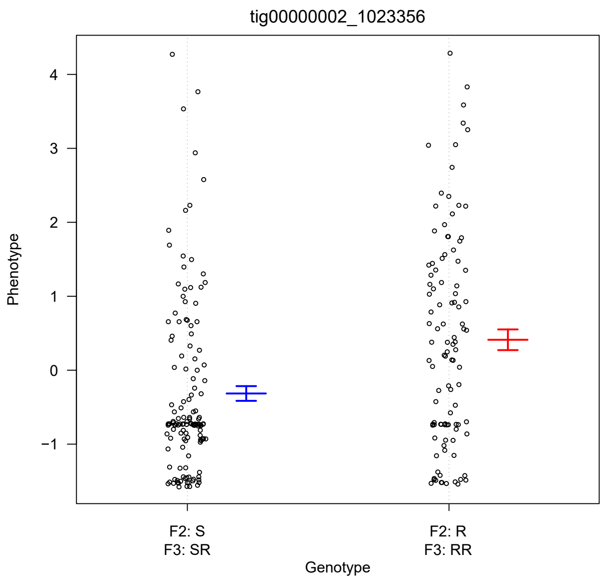

Figure S3. Phenotype x genotype interaction at the peak marker location. Corrected F3 phenotype observations (y-axis) are grouped by the genotype of their F2 father (x-axis) at the marker location with the highest LOD score for the nonparametric QTL model. High phenotype values indicate high reproductive success of F3 individuals on *Hamiltonella* protected hosts. Genotype S corresponds to the susceptible population origin (maternal genotype in the P generation) and genotype R corresponds to the resistant population origin (paternal genotype in the P generation). F2 genotpyes were sequenced, F3 genotypes were inferred from F3 genotypes. Mean ± SEM corrected phenotype values are shown in blue and red for the maternal and paternal genotype, respectively.

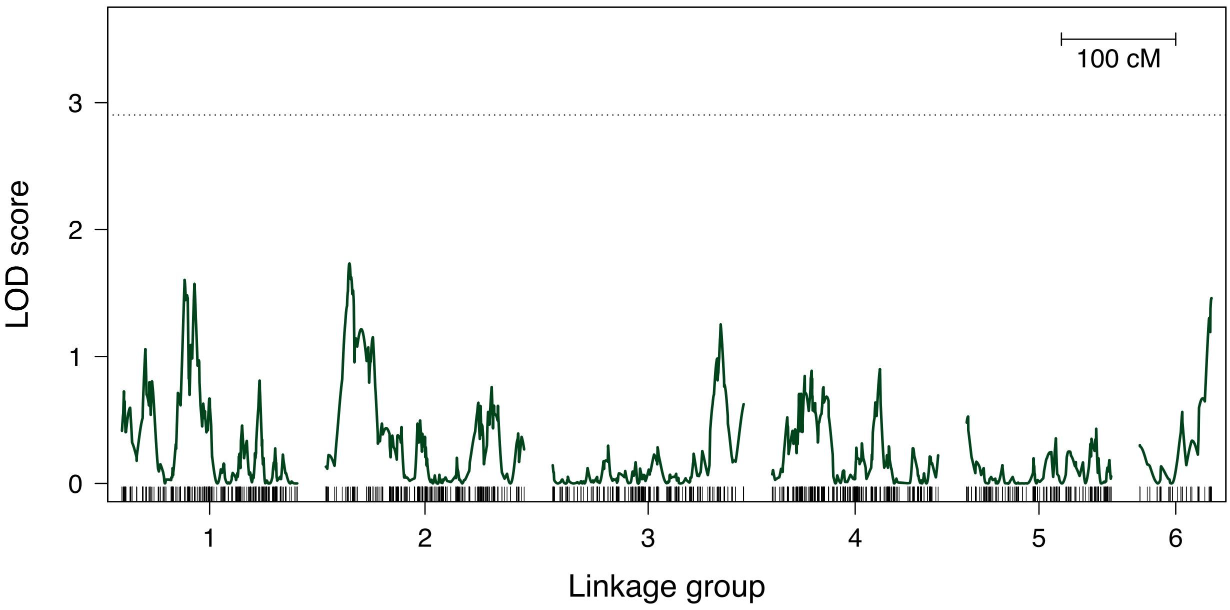

Figure S4. **Additional QTL mapping with binary phenotype.** LOD scores of the binary QTL model do not show any significant QTLs. The phenotypic measure of counteradaptation was a binary variable indicating whether offspring was observed on *Hamiltonella*-protected hosts. The horizontal dotted line shows the global significance threshold for $P = 0.05$, determined by permutation test (N replicates = 1000). Vertical ticks at the bottom show marker locations for each linkage group.
